# The importance of environmental microbes for *Drosophila melanogaster* during seasonal macronutrient variability

**DOI:** 10.1038/s41598-021-98119-0

**Published:** 2021-09-22

**Authors:** Lucy Rebecca Davies, Volker Loeschcke, Mads F. Schou, Andreas Schramm, Torsten N. Kristensen

**Affiliations:** 1 Department of Biology, Aarhus University, 8000 Aarhus, Denmark; 2 Department of Biological and Environmental Science, University of Jyväskylä, 40500 Jyväskylä, Finland; 3 Department of Biology, Lund University, 223 62 Lund, Sweden; 4 Department of Chemistry and Bioscience, Aalborg University, 9220 Aalborg, Denmark

**Keywords:** Ecology, Evolution

## Abstract

Experiments manipulating the nutritional environment and the associated microbiome of animals have demonstrated their importance for key fitness components. However, there is little information on how macronutrient composition and bacterial communities in natural food sources vary across seasons in nature and on how these factors affect the fitness components of insects. In this study, diet samples from an orchard compost heap, which is a natural habitat for many *Drosophila* species and other arthropods, were collected over 9 months covering all seasons in a temperate climate. We developed *D. melanogaster* on diet samples and investigated stress resistance and life-history traits as well as the microbial community of flies and compost. Nutrient and microbial community analysis of the diet samples showed marked differences in macronutrient composition and microbial community across seasons. However, except for the duration of development on these diet samples and Critical Thermal maximum, fly stress resistance and life-history traits were unaffected. The resulting differences in the fly microbial community were also more stable and less diverse than the microbial community of the diet samples. Our study suggests that when *D. melanogaster* are exposed to a vastly varying nutritional environment with a rich, diverse microbial community, the detrimental consequences of an unfavourable macronutrient composition are offset by the complex interactions between microbes and nutrients.

## Introduction

Organisms are associated with a vast amount of commensal bacteria that can support metabolic signalling and nutrient processing. These microbes can synthesize essential vitamins and impact energy harvesting and fat storage of the host^1–3^. In times of nutrient deficiencies, microbes can also modulate growth signaling pathways to restore growth when ingested nutrients are inadequate for this process^4^.

Most organisms inhabit environments where nutrient availability fluctuates in space and time and may therefore depend on their associated microbes for survival and growth. As compatible microbes from the environment can establish themselves within the host through food ingested, the composition and abundance of these microbes can vary according to the host’s environment^5,6^. For example, several studies on species of *Drosophila* show considerable temporal and spatial variation in their associated microbiota^5–9^. Associations with higher taxonomic categories, such as *Acetobacteracae*, *Enterobacteriaceae* and *Lactobacillaes,* are repeatedly seen in these investigations, but differences in the abundance of genera and species are found across environments^5–8^.

Organisms face natural changes in abiotic factors in their environments, such as temperature and humidity, across days, seasons and localities, which will mediate changes in the microbial community and macronutrient availability^10,11^. It is well documented that varying the microbial community and macronutrient availability have large fitness impacts in some *Drosophila* species^5,12–18^. For example, *D. melanogaster* males monoinfected with *Acetobacter pomorum* induce low offspring production in females compared to the high offspring production of females mated with *Lactobacillus plantarum-*infected males^19^. Furthermore, growth levels of *D. melanogaster* larvae on low-protein diets associated with increased densities of *A. pomorum* or *L. plantarum* corresponded to the much higher growth levels normally observed on high-protein diets^16,18^.

Variations in protein and carbohydrate availability induce significant changes in *Drosophila* fitness^13,14,17^. For instance, adult *D. melanogaster* live longer on high carbohydrate diets but have lower reproductive performance compared to those on a low carbohydrate, high protein diet^13,14,17^. However, organisms can sense these variations and respond by changing their foraging choices to maximise their performance^13,17,20^, such as increasing the intake of a protein source when protein levels are low^12,13^. Wong et al*.*^21^ found that associated microbes can also influence these foraging behaviours. *L. plantarum*-inoculated *D. melanogaster* preferred a diet high in carbohydrates, for example. Such results suggest that, in addition to the associations between organisms and microbes and organisms and nutrients, interactions between nutrients and microbes can occur and have consequences for organism fitness.

In addition to nutrient-microbe interactions, temperature can influence the phenotypic response to different nutrients and vice versa. An increased developmental temperature was found to worsen the negative consequences of a high carbohydrate diet, and high protein availability was shown to be beneficial for the ability of *D. melanogaster* to tolerate heat and cold stress^22,23^. The diet and microbial community may therefore be crucial components for the optimization of fitness as seasonal environmental conditions change in nature.

Due to their significant effects on phenotypic traits, investigating interactions between multiple environmental variables is important when assessing organisms’ responses to environmental change. Most investigations studying the impacts of microbes on fitness components have been conducted using artificial diets inoculated with a defined cocktail of microbes. Here, we assessed the bacterial composition of the microbiota and the nutritional content of a natural food source composed of decomposing fruit from a compost heap sampled across a nine-month period from May 2014 to January 2015. Second, we fed the nine diet samples to *D. melanogaster* and investigated several stress resistance and life-history traits and the bacterial composition of flies’ microbiota exposed to natural food sources. With this setup, we tested (1) whether protein and carbohydrate abundance and the microbial community of the diet samples varied across seasons; (2) how any seasonal change in nutrition and microbial community in the compost affected the established microbiome of the flies; and (3) whether changes in the nutritional and microbial composition of diets across seasons resulted in adaptive changes in fitness components of the flies.

## Results

### Microbial community structure of diet samples

The diet microbiotas were dominated by a few genera, such as *Acetobacter*, *Lactobacillus*, *Gluconobacter* and *Brevundimonas* (Supplementary Fig. S1A).

**Figure 1:**
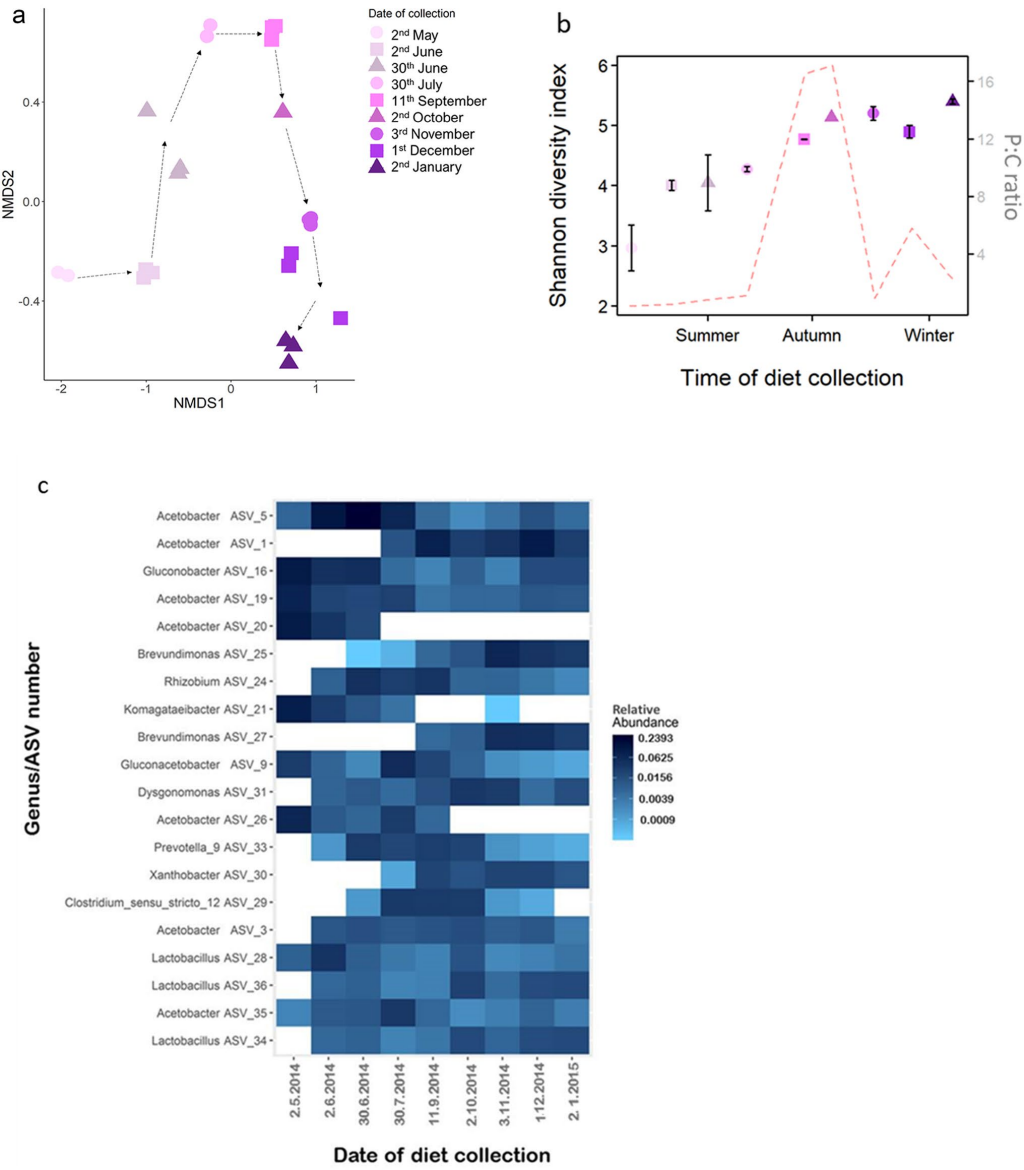
Nutritional composition and microbial community structure of the natural diets (*n* = 1–3). (**a**) Two-dimensional nonmetric multidimensional scaling (NMDS) axes based on Bray–Curtis dissimilarity matrices of amplicon sequence variants (ASVs). Each point represents the microbial community in a replicate. Dotted arrows inserted for easy interpretation of associations with date of collection. (**b**) Shannon diversity index (left y-axis) of the microbial community of the diet samples across the collection dates. Points show mean (± 95% CI). Colour and symbol code same as (**a**). The red dotted line represents the protein to carbohydrate (P:C) ratio (right y-axis). (**c**) The 20 relatively most abundant ASVs across diet samples with relative abundance indicated by the colour coding. Figures (**a,b**) created using R package ggplot2 v3.3.3 (https://ggplot2.tidyverse.org/). Figure (**b**) created using R package phyloseq v1.34.0 (https://github.com/joey711/phyloseq).

Nonmetric multidimensional scaling (NMDS) analysis based on Bray–Curtis dissimilarity matrices of amplicon sequence variants (ASVs) showed clear differences in the microbial community structure among diets (Fig. 1a). This was confirmed by an ANOSIM test (R = 0.98, *P* < 0.001). Underlying this difference in microbial community structure was an influence of the protein to carbohydrate (P:C) ratio (Mantel test: r = 0.15, P < 0.05) as well as an influence of the average temperature of the month the diets were collected (Mantel test: r = 0.49, *P* < 0.001). Using the Shannon diversity measure, we found that the bacterial diversity of the diets was significantly different across the collection days (*F*_1,22_ = 41.78, P < 0.001) (Fig. 1b). The diversity of the diet collected on 2nd May was significantly lower than that of all the other diets, while the diversity of the diets collected in late autumn to winter was significantly higher than that of those collected in summer (Fig. 1b, Supplementary Tables S2, S3).

In the top 20 most abundant ASVs present, we found that the relative abundance of eight microbial strains significantly decreased as the year progressed; seven significantly increased, and the remaining microbial strains did not change significantly (Fig. 1c, Supplementary Table S4).

### Microbial community structure of fly samples

**Figure 2:**
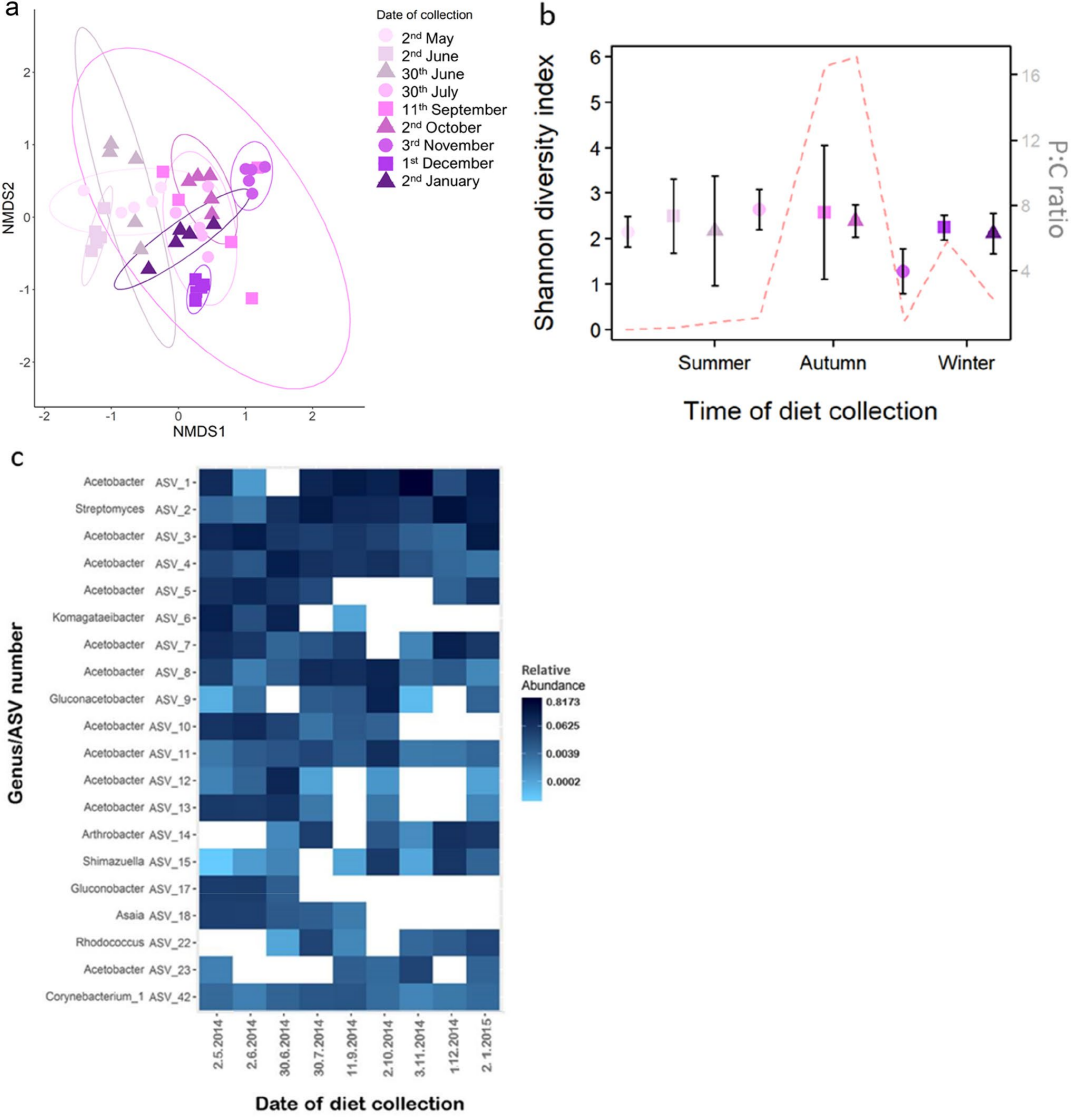
Microbial community structure of the flies developed on the different diets (*n* = 4–5). (**a**) Two-dimensional nonmetric multidimensional scaling (NMDS) axes based on Bray–Curtis dissimilarity matrices of amplicon sequence variants (ASVs). Each point represents the microbial community in a sample. Flies were grouped in 5 to represent one sample. (**b**) Shannon diversity index (left y-axis) of the microbial community of the fly samples across the food collection days. Points show mean (± 95% CI). Smaller points represent replicates. Colour and symbol code same as (**a**). The red dotted line represents the protein to carbohydrate (P:C) ratio of the diet (right y-axis). (**c**) The 20 relatively most abundant ASVs across fly samples with relative abundance indicated by the colour coding. Figures (**a,b**) created using R package ggplot2 v3.3.3 (https://ggplot2.tidyverse.org/). Figure (**b**) created using R package phyloseq v1.34.0 (https://github.com/joey711/phyloseq).

The microbiotas of the flies were highly dominated by the genus *Acetobacter* (Supplementary Fig. S1B) and differed significantly across the diets they consumed (ANOSIM test: R = 0.76, *P* < 0.001, Fig. 2a), although this distinction was not as strong as for the microbial community structure of the diet samples. Nevertheless, we still detected an influence of the P:C ratio of the diet on the microbiota of the flies (Mantel test: R = 0.13, *P* < 0.001) and the average monthly temperature when samples were collected (Mantel test: R = 0.16, *P* < 0.01). The Shannon diversity of the microbiota of flies did not differ across the collection day of the diets (*F*_1,44_ = 1.07, *P* = 0.4), nor did it differ across the P:C ratios of the diet (*F*_1,44_ = 1.09, *P* = 0.3 (Fig. 2b, Supplementary Table S2).

The Shannon diversity of the microbiota of the flies was significantly lower than that of the diets (*F*_1,67_ = 108.58, P < 0.001, Fig. 2b, Supplementary Table S2). The microbiota of the flies was also significantly different from that of their diets (ANOSIM test: R = 0.64, *P* < 0.001, Supplementary Fig. S2).

As with the diet samples, we next took a closer look at the top 20 relatively most abundant ASVs. Half of the top microbial strains did not change significantly across the collection dates (Fig. 2c, Supplementary Table S4). The remaining ASVs showed variations depending on the collection date of the diet (Supplementary Table S4).

Finally, we assessed the share of ASVs between the flies and their respective diets. Based on Sørensen’s dissimilarity measure^24^, the microbiome of the flies was significantly different from that of the diet samples (ADONIS: R^2^ = 0.15, P < 0.001) (Supplementary Fig. S2). By merging the fly samples with their respective diet and creating nine groupings, we found that 3–17% of the total ASVs in each diet group were shared (Table 2); 20% of these were found in at least two of the fly diet groups (Supplementary Table S5).

### Impact of diet on fly fitness components

**Figure 3:**
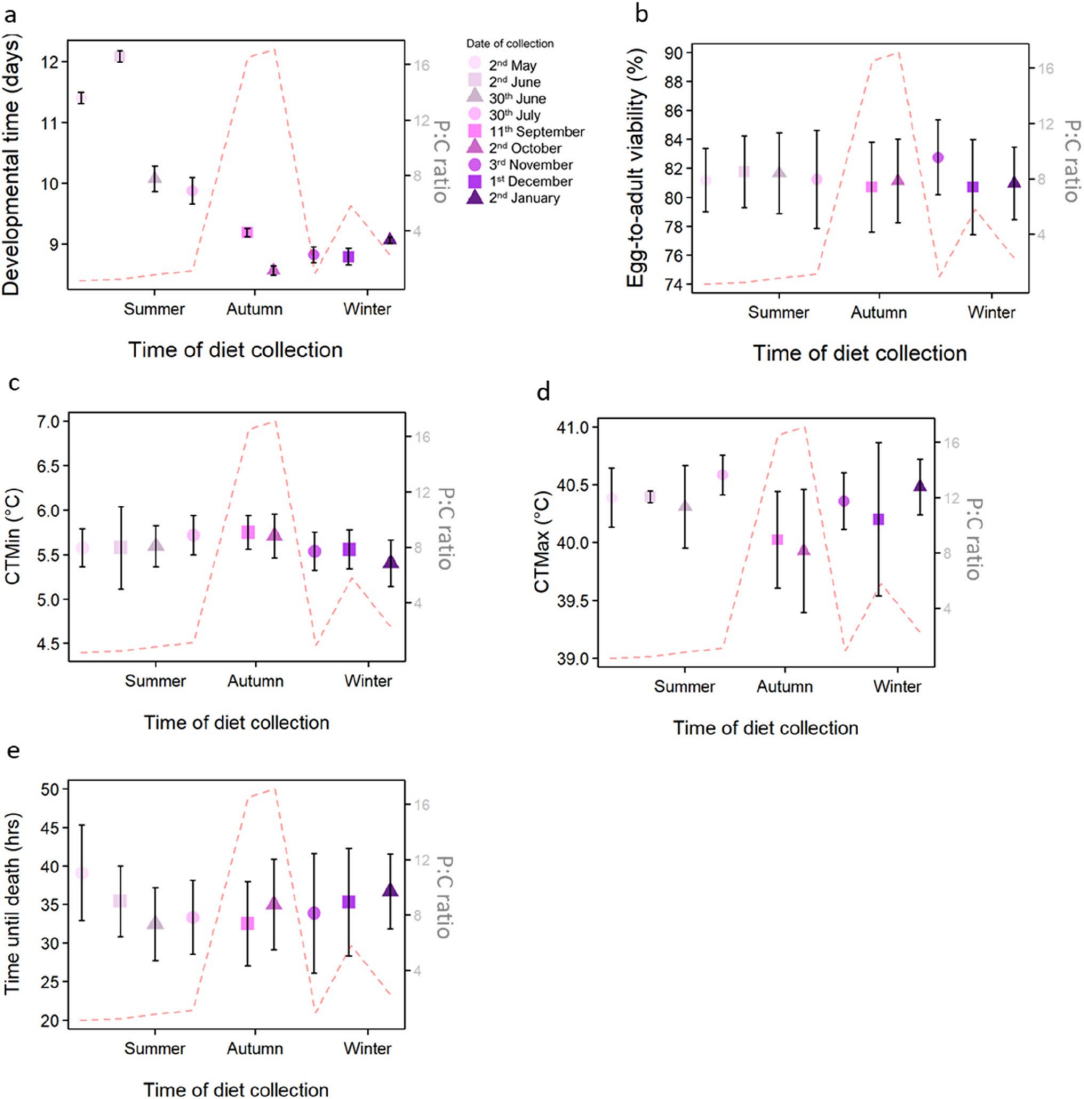
Results from the phenotypic assessments. Points show mean (± 95% CI). Colour and symbol code same as (**a**) for all figures. Phenotypic trait assessed shown on the left y-axis. The red dotted line represents the protein to carbohydrate (P:C) ratio of the diet (right y-axis). (**a**) Developmental time (*n* = 100–120). (**b**) Egg-to-adult viability (*n* = 17–19 vials). (**c**) Critical Thermal minimum (CTmin) (*n* = 13–17). (**d**) Critical Thermal maximum (CTmax) (*n* = 12–17). (**e**) Starvation resistance (*n* = 14–18). All figures created using R package ggplot2 v3.3.3 (https://ggplot2.tidyverse.org/).

The developmental time across the natural diets was significantly different (*F*_1,100_ = 184.5, *P* < 0.001). Flies developed on diets collected in summer had significantly longer developmental time, with flies developing on the diet collected on June 2nd having the longest developmental time (Fig. 3a, Supplementary Table S4). Developmental time did not significantly differ between flies developed on diets collected in late Autumn (Fig. 3a, Supplementary Table S6). Developmental time was negatively correlated with the P:C ratios of the diets (*F*_1,100_ = 34.78, *P* < 0.001) (Fig. 3a).

Conversely, flies maintained constant egg-to-adult viability across the diets from different seasons (χ^2^ = 166.63, *P* = 0.99) and P:C ratios (χ^2^ = 166.63, *P* = 0.53) (Fig. 3b). There was also no significant difference between the Critical Thermal minimum (CTmin) of the flies (*F*_1,126_ = 0.62, *P* = 0.75) (Fig. 3c). The sensitivity of the flies to low temperatures appeared to have a peak when the P:C ratio was at its highest (Fig. 3c); however, this was not statistically significant (*F*_1,126_ = 1.98, *P* = 0.16). In contrast, for tolerance towards high temperatures, the Critical Thermal maximum (CTmax) appeared to decrease when the P:C ratio was high, and this negative correlation was statistically significant (*F*_1,131_ = 8.26, *P* < 0.05) (Fig. 3d). There was, however, no significant difference between the diet collection times (*F*_1,131_ = 1.22, *P* = 0.27) (Fig. 3d).

No significant difference was found in starvation resistance across the collection time (*F*_1,149_ = 0.56, *P* = 0.82), and there was no correlation with the P:C ratio (*F*_1,149_ = 0.32, *P* = 0.57) (Fig. 3e).

## Discussion

### Changes in diet composition throughout the year

In nature, *D. melanogaster* acquires much of their carbohydrate and protein from decaying fruit and yeasts growing on the fruit^25^. During fruit decay, yeasts break down the fruit tissue and its sugar components, which should cause temporal fluctuations in the amount of protein and carbohydrate available to other organisms^10^. In diets collected in late spring to summer, carbohydrate was more prevalent than protein. This changed in the colder months, where protein levels increased and carbohydrate levels decreased, but only until November, where protein levels decreased again, probably due to the fruit being too decomposed for yeasts to successfully metabolize it (Table 1).

**Table 1:** Natural diet sample information: collection day (counted from the date of the first sample collected), date of sample collection, average temperature of the month the diets were collected, protein value, carbohydrate value and protein to carbohydrate ratio of each sample.

Collection day	Date of collection	Average monthly temperature (°C)	Protein (g/100 g)	Carbohydrate (g/100 g)	P:C ratio
1	2.5.2014	11.6	1.14	2.9	0.4
32	2.6.2014	14.7	1.35	2.7	0.5
60	30.6.2014	14.7	1.40	1.8	0.8
90	30.7.2014	19.1	1.41	1.3	1.1
134	11.9.2014	14.3	1.65	0.1	16.5
155	2.10.2014	12	1.71	0.1	17.1
187	3.11.2014	7.6	1.89	2	0.9
215	1.12.2014	3.2	1.73	0.3	5.8
248	2.1.2015	2.8	1.42	0.7	2.0

We also found very distinct changes in microbial community structures of the natural diet samples collected across the year (Fig. 1a). The change in relative abundance of some of the most prevalent ASVs, *Gluconobacter*, *Acetobacter* and *Komagataeibacter*, is consistent with these genera’s preference for carbohydrate-rich environments^26–29^. Thus, their decrease in relative abundance followed the decrease in carbohydrate in the Danish winter (Fig. 1c). This illustrates that throughout the year, flies (and other arthropods affiliated with compost heaps) are exposed not only to nutritional fluctuations but also to corollary changes in the microbial community structure of their diet.

### Characteristic differences between microbial communities in diets and flies

The microbial diversity and the most abundant genera of the flies differed from those of the diet samples (Fig. 1c, 2c, Supplementary Fig. S3). The top 20 ASVs found in the flies were dominated by ASVs from *Acetobacter* (Fig. 2c, Supplementary Fig. S1B) and ASVs not found in the diet samples (Supplementary Fig. S3). *D. melanogaster* obtains most of its microbiota from bacteria on rotting fruit and faecal matter in their environment or inherits it via egg surfaces^30–34^. As eggs from our laboratory stock were not washed when transferred to the diet samples, the microbial community structure of the flies was a mix of compatible microbes from the diet and the laboratory stock.

We found only a small number of microbes that were shared between the diet and the fly samples (Table 2, Supplementary Table S5). Even fewer microbes were shared as the year progressed, and the diversity of the diet samples increased (Fig. 1a, Table 2), which could be a result of several factors. A number of physiological and immune response features of the fly gut are known to contribute to microbial regulation^35^. Only a small number of taxa can therefore survive in the gut of *Drosophila* species^5,34,36^. This active regulation of residing microbes would also explain the overall lower diversity in our fly samples compared to the diets (Fig. 2b, Supplementary Table S2).

**Table 2:** The distribution of ASVs when the fly samples are grouped with their respective diet.

Collection day of diet	Date of collection	Number (%) shared ASVs	Number (%) ASVs belonging to diet sample only	Number (%) ASVs belonging to flies only
1	2.5.2014	92 (17)	166 (31)	279 (52)
32	2.6.2014	98 (11)	305 (36)	453 (53)
60	30.6.2014	94 (11)	322 (35)	492 (54)
90	30.7.2014	61 (9)	307 (47)	284 (44)
134	11.9.2014	59 (7)	460 (54)	326 (39)
155	2.10.2014	41 (8)	265 (52)	204 (40)
187	3.11.2014	70 (8)	509 (59)	287 (33)
215	1.12.2014	51 (7)	531 (58)	261 (35)
248	2.1.2015	28 (3)	676 (74)	212 (23)

The percentage of the total ASV counts is shown in brackets.

Interestingly, we found abundant *Streptomyces* in all the fly samples but only a trace abundance in just one of the diet samples (Supplementary Fig. S3, Fig. 2b). As *Streptomyces* is usually found in soil and has a high growth rate at warmer temperatures^37^, we found it likely that *Streptomyces* was present in all diet samples in trace amounts but that the relatively high temperature of the laboratory experiments resulted in an exponential increase during exposure to flies. However, we have found no evidence in the literature of *Streptomyces* being a successful symbiont of *Drosophila*, but one explanation could be that its presence is transient. This result also highlights that the microbial community of the diet may have changed during the development of the larvae, which could have also resulted in a change in the microbial load of the food. An analysis of the diet after the larval phase could have confirmed this.

### Fitness components are constant despite macronutrient fluctuations of the diet

The developmental time of the flies was the only trait significantly impacted by the collection time of the fly diet (Fig. 3a, Supplementary Table S5). Apart from developmental time and CTmax, none of the other traits significantly correlated with the P:C ratio of the diet (Fig. 3d). This was unexpected, as changes in protein and carbohydrate amounts in the diet during laboratory experiments are known to influence a range of traits in insects^13,17,38–41^. This is also the case in *D. melanogaster*^16^, where low protein content in the diet leads to increased egg-to-adult viability and starvation^17^, and higher protein content leads to increased heat and cold tolerances^21^. In contrast to our findings, we therefore expected the fluctuating protein and carbohydrate contents in the nine natural diets to influence all traits. One reason for this discrepancy could be the use of highly simplified foods with very little microbial diversity in previous laboratory investigations. We suggest that the diverse microbes associated with natural diets play an important role in reducing the impact of nutritional fluctuations on fly phenotypes, and we discuss this further below.

### Bacteria to the rescue

The relationship between resident microbes and host physiology is complex and influenced by a range of factors, such as host genotype^42^, immunity^43^ and diet^44^. For *D. melanogaster,* we know that although an associated microbial community is not essential for survival, it can be extremely beneficial in times of macronutrient stress^4,15,18,45^. Association with certain species of *Acetobacter*, for example, can also alter the nutritional balance of the food ingested by *D. melanogaster,* as these microbes can selectively consume sugars, preventing the fly from digesting too much carbohydrate^3^. We therefore speculate that the high relative abundance of *Acetobacter* in both the diets and the flies in the present study (Supplementary Fig. S1A,B) had a balancing effect on food ingestion when carbohydrate levels were high in the warmer months, stabilizing fitness components of flies across the year. This conclusion, however, must be made with caution: Keebaugh et al*.*^18^ also showed that diets with higher amounts of yeast can provide ideal growth conditions for bacteria^18^, so even though the relative abundance of *Acetobacter* is high during high carbohydrate periods (Fig. 1b, Supplementary Fig. S1A,B), the absolute amount of *Aceobacter* could be exactly the same during colder months. Our results find a positive correlation with the P:C ratio and the diversity of natural food (Fig. 1b), which supports Keebaugh et al.^18^.

Additionally, flies developed on an unfavourable, imbalanced diet have increased fitness when the diet is supplemented with high amounts of bacteria^18,46^. Keebaugh et al*.*^18^ showed that the presence of sufficient quantities of *Acetobacter* species can ameliorate the negative effects of insufficient protein on lifespan and larval viability. Developing on a microbial rich, natural diet could thus be beneficial for an organism going through macronutrient stresses, although it is unclear if the microbes are a valuable food source or if they provide crucial amino acids^47,48^.

## Conclusion

Artificial laboratory diets where macronutrient amounts are manipulated have provided important insights into the phenotypic responses to varying macronutrients. Sterile practices, however, of autoclaving food and regularly providing fresh food reduce the microbial diversity in the food and compromises the ecological relevance of the diet. Here we quantified the natural fluctuations in macronutrient availability and microbial community structure that *D. melanogaster* faces in nature. The investigated fitness components were largely unaffected by these natural fluctuations. Because of the complex nature of microbe-nutrient-organism interactions though, many factors could play a role in this phenotypic canalization. To fully characterise the importance of these factors we therefore advocate future work sterilizing the natural diet to separate the effects of environmental microbes and the natural diet.

This study provides an example where varying the protein to carbohydrate ratios does not significantly affect phenotypic traits known to be influenced by diet. Based on our results, we suggest that a diet made up of a diverse range of microbes could offset the detrimental effects of an unfavourable macronutrient composition without directly impacting the associated microbiota of the fly.

## Methods

### Origin of flies

The *D. melanogaster* population used in this study was from a laboratory stock established from the offspring of approximately 600 inseminated females caught near Odder (55° 56′ 42.46″ N, 10° 12′ 45.31″ E), Denmark, in 2010. The flies were maintained on a 12 h:12 h light:dark cycle at 19 °C on standard laboratory food composed of yeast (60 g/L water), sucrose (40 g/L water), oatmeal (30 g/L water), and agar (16 g/L water) mixed with tap water. Following autoclaving, nipagin (12 mL/L water) and acetic acid (1.2 mL/L water) were added.

Prior to the experiment, flies were taken from the stock and kept at 25 °C for three generations, the temperature at which the experiment was conducted. Population density was controlled by transferring 30 eggs to vials containing 7 mL of standard laboratory medium. Once emerged, 200 adults were transferred to bottles containing 35 mL of standard laboratory *Drosophila* medium and left to lay eggs overnight. This was done for three generations.

After these three generations, the experiment was initiated by transferring 40 eggs laid on medium in 35 mL bottles to vials containing 7 mL of our experimental diets. This was done across 5 × 8 h periods to account for any differences in developmental time^49^. The use of laboratory-reared flies ensured a more similar microbiome across individuals with low diversity^5,32,36^.

### Natural diets

The natural diets originated from a compost heap at the orchard where the laboratory stock flies were collected, Odder (55° 56′ 42.46″ N, 10° 12′ 45.31″ E), Denmark. The compost heap was established based on windfall apples and pears collected in November 2013. Nine samples (of ca. 1.5 kg each) were collected from the heap across nine months in 2014 (Table 1). Each sample was made up of collections taken from multiple areas of the heap due to the variability of materials in the heap. The samples were stored at − 20 °C, whereby changes in microbial community and microbial viability over the period of storage time would be very minor^50,51^. Before measurements were taken, the diets were blended. The average monthly temperatures of the location of the collected samples were obtained from the Danish Meteorological Institute, www.dmi.dk (Table 1).

During pilot experiments, we observed dominant fungal growth in the natural food when kept at 25 °C in the lab, which significantly impacted the development and survival of the flies. We therefore added nipagin (12 mL/L) to the compost to solve this problem before exposing flies to the diets. The low concentrations have been shown not to change the viability of the bacterial community or affect gut bacterial composition directly^52–55^.

### Nutritional composition of natural diets

The nutritional compositions of the natural diets were analysed by ALS Food and Pharmaceutical (http://www.alsglobal.com/) using the methods described in Supplementary materials Table S1. Protein and carbohydrate (not including indigestible fibre) amounts are displayed in Table 1.

### Microbiota molecular procedure and processing of sequence data

DNA extraction and sequencing were performed externally by DNASense, Aalborg, Denmark (http://www.dnasense.com). The outside of the flies was washed with ethanol before being sent for sequencing. DNA was extracted from blended samples of the natural diets and whole flies pooled in groups of five. Three diet-free and fly-free negative controls were also included. The extractions were performed using the DNeasy Blood and Tissue kit according to the manufacturer’s protocol (Qiagen, Germany).

The V1–V3 region of the bacterial 16S rRNA gene was amplified in duplicate per sample using primers 27F AGAGTTTGATCCTGGCTCAG and 534R ATTACCGCGGCTGCTGG^56^. Duplicate PCR products were pooled for library construction according to Caporaso et al.^57^ and were paired-end sequenced (2 × 300 bp) on a MiSeq using Reagent kit v3 following the standard protocol (Illumina, USA). Three diet replicates and five replicates of fly groups from each collection date were sequenced. However, not all samples produced sufficient sequencing depth and reads for analysis (*n* of replicates: 2nd May: diet = 2, fly = 5; 2nd June: diet = 3, fly = 5; 30th June: diet = 3, fly = 4; 30th July: diet = 2, fly = 5; 11th Sept: diet = 3, fly = 5; 2nd Oct: diet = 1, fly = 5; 3rd Nov: diet = 3, fly = 5; 1st Dec: diet = 3, fly = 5; 2nd Jan: diet = 3, fly = 5).

The DADA2 pipeline (which can be found at https://github.com/benjjneb/dada2) processes Illumina MiSeq raw amplicon sequences into a table of amplicon sequence variants (ASVs), a higher-resolution measure than OTU clustering^58^, which are present and the number of times each ASV is observed in each sample. To obtain our ASV table, we followed the pipeline using the DADA2 “standard” filtering and trimming parameters (https://github.com/benjjneb/dada2)^58^, adjusting only the truncation length to 280 and 260 to suit our chosen primers. The core DADA2 algorithm^58^ was then applied to the filtered and trimmed sequence data, and chimeras were removed from the ASV table. Taxonomy was then assigned using the Silva version 132 database^59^. An average of 18,059 reads per diet sample and an average of 24,347 reads per fly sample were assigned to bacterial ASVs (Supplementary Table S2).

### Phenotypic assessment

Developmental time, egg-to-adult viability, starvation resistance and Critical Thermal maximum (CTmax) and minimum (CTmin) were assessed. Developmental time was assessed by counting flies that had emerged in each vial every 12 h (40 eggs per vial, *n* of vials: 2nd May = 10, 2nd June = 14, 30th June = 9, 30th July = 12, 11th Sept = 13, 2nd Oct = 11, 3rd Nov = 9, 1st Dec = 11, 2nd Jan = 12). Egg-to-adult viability was measured as the percentage of flies that emerged from the 40 eggs in each replicate vial across the nine developmental diets (*n* of vials: 2nd May = 19, 2nd June = 17, 30th June = 18, 30th July = 18, 11th Sept = 18, 2nd Oct = 18, 3rd Nov = 18, 1st Dec = 18, 2nd Jan = 18). On the day starvation resistance and thermal tolerances were measured, at 00:00, emerged flies were counted and removed. Male flies that had emerged between 0:00 and 8:00 were used for the stress resistance tests. Therefore, at 8:00, the males were collected (by sight, with no CO_2_) and randomly assigned to a stress resistance experiment. Starvation resistance was measured by placing one fly per vial that contained 3.5 mL of water and agar (2%) and counting how many flies died every 8 h (*n* of flies: 2nd May = 18, 2nd June = 14, 30th June = 17, 30th July = 18, 11th Sept = 16, 2nd Oct = 16, 3rd Nov = 17, 1st Dec = 17, 2nd Jan = 17). CTmax was measured by methods described in Overgaard, Kristensen and Sørensen^60^. In short, we placed flies from each diet in sealed 5 mL screw cap glass vials with one fly per vial. These vials were then fixed onto racks and immersed in a water tank. The temperature of the water then increased from 25 by 0.1 °C per minute. The CTmax score was the temperature of the water when the fly stopped responding by physical movement to light and prodding of the vial with a metal stick (*n* of flies: 2nd May = 15, 2nd June = 16, 30th June = 16, 30th July = 13, 11th Sept = 16, 2nd Oct = 13, 3rd Nov = 17, 1st Dec = 14, 2nd Jan = 12). CTmin was measured using the same setup but with the temperature decreasing by 0.1 °C per minute (*n* of flies: 2nd May = 14, 2nd June = 16, 30th June = 13, 30th July = 15, 11th Sept = 17, 2nd Oct = 14, 3rd Nov = 13, 1st Dec = 13, 2nd Jan = 13).

### Statistical analysis

Statistical analysis was performed using R software v4.0.4^61^ in R studio v1.4.1106^62^. Analyses of the microbial communities were performed on the relative abundance of each ASV using the phyloseq v1.34.0 package in R^63^. Patterns of dissimilarities were visualized using nonmetric multidimensional scaling (NMDS) using Bray–Curtis dissimilarity. Further statistical analysis of the dissimilarities within the microbial communities of the diet samples and within the fly samples was performed using the R package vegan v.2.5-7^64^. First, we conducted ANOSIM tests to determine whether the microbial communities within the diet samples and within the fly samples were significantly different. This was followed by Mantel tests, based on Bray–Curtis dissimilarity matrices of the ASVs, to calculate the correlations between the microbial communities and the P:C ratio of the diet and the average temperature of the month the diets were collected. Collection day was additionally used as a continuous variable to conduct Pearson’s correlation tests conducted on individual ASVs to investigate their change throughout the year.

For the statistical analyses on diversity measures and phenotypic assessments, the day of collection of the diets (Table 1) was used as the predictor variable and was considered a factorial variable. Statistical analyses were also performed on diversity measures and phenotypic assessments using the P:C ratios of the diets (Table 1) as a continuous variable.

Correlations between carbohydrate amount, measures of microbial diversity and relative abundance of certain bacteria were assessed using Pearson’s correlation coefficient. For protein amount, a linear model containing protein amount as the response variable was compared with a quadratic model using an F test to obtain the *P*-value.

The effect of the collection day of the developmental diet on developmental time, CTmin, CTmax and starvation resistance was assessed by constructing separate linear models. The models contained the average developmental time of each vial, CTmin, CTmax or starvation resistance as the response variable. These models were compared to a reduced model using an F test to obtain the *P*-values.

To investigate the effect of the collection day of the diet on egg-to-adult viability, we used logistic regression in a generalized linear model. The model contained the day of collection as the only predictor variable. We detected no over-dispersion in the model. We compared the full model with a reduced model using a likelihood ratio test to obtain a *P*-value for the effect of collection day of the diet.

All figures were produced using the ggplot2 R package v3.3.3^65^, except for heatmaps, which were produced using the phyloseq v1.34.0 package^63^.

## Supplementary Information

Supplementary Information.

## Data Availability

All data can be found at https://figshare.com/projects/The_importance_of_environmental_microbes_for_Drosophila_melanogaster_during_seasonal_macronutrient_imbalances/85625.
