# Supplementary material for: The importance of environmental microbes for Drosophila melanogaster during seasonal macronutrient variability
Source: Sci Rep. 2021 Sep 22;11:18850. doi: 10.1038/s41598-021-98119-0 (PMC8458401; doi:10.1038/s41598-021-98119-0)

**The importance of environmental microbes for *Drosophila melanogaster* during seasonal macronutrient variability**

Lucy Rebecca Davies* ^a,b^, Volker Loeschcke ^a^, Mads F. Schou ^a,c^, Andreas Schramm ^a^ and Torsten N. Kristensen ^d^

^a^ Department of Biology, Aarhus University, DK-8000 Aarhus, Denmark

^b^ Department of Biological and Environmental Science, University of Jyväskylä, Jyväskylä, 40500, Finland

^c^ Department of Biology, Lund University, Lund, 223 62, Sweden

^d^ Department of Chemistry and Bioscience, Aalborg University, DK-9220 Aalborg, Denmark

*Corresponding author: [lucy.r.davies@jyu.fi](mailto:lucy.r.davies@jyu.fi)

**Supplementary material**

**Supplementary table S1.** Methods used to analyse nutrient content of the natural diet samples.

**Supplementary table S2.** Number of reads after filtering steps and alpha diversity measures of the microbial community of diet and fly samples.

**Supplementary table S3.** Tukey post hoc comparisons for the Shannon diversity of the diet samples.

**Supplementary table S4.** Pearson’s coefficient values (r^2^) for correlations between the top 20 genera relative abundance and day of diet sample collection. * indicates significant correlation where *P* < 0.05, ** indicates significant correlation where *P* < 0.01 and *** indicates significant correlation where *P* < 0.001.

**Supplementary table S5.** List of ASVs and their genus that are found in the microbiome of at least two groups made up of flies and their respective diet.

**Supplementary table S6.** Tukey post hoc comparisons for the developmental time.

**Supplementary Figure S1.** Stacked bar graphs of the top 12 and “other” genera. (A) Diet samples and (B) Fly samples. Figures created using R package phyloseq v1.34.0 (https://github.com/joey711/phyloseq).

**Supplementary Figure S2.** Microbial community structure of the diet samples and the flies developed on the different diets using a two-dimensional nonmetric multidimensional scaling (NMDS) axes based on Bray-Curtis dissimilarity matrices of amplicon sequence variants (ASVs). Each point represents the microbial community in a specific sample. Figure created using R package ggplot2 v3.3.3 (<https://ggplot2.tidyverse.org/>).

**Supplementary Figure S3.** Heatmap showing how the patterns of the 20 most relatively abundant ASVs found in the fly samples looks in the diet samples. Figure created using R package phyloseq v1.34.0 (https://github.com/joey711/phyloseq).

**Supplementary table S1.**

| **Analysis** | **ALS method reference** | **Standard reference** | **Method summary** |
| --- | --- | --- | --- |
| Moisture (loss on drying) | AM/C/801 AM/C/101 5 | AOAC 16th Edition Feeding Stuffs Sampling & Analysis Regulations 2000 | The sample is dried in an oven at a specified temperature. The loss in weight is equivalent to the moisture content (loss on drying) and is expressed as a percentage of the sample. |
| Nitrogen protein (N x 6.25) | AM/C/224 | Dumas Leco or Elementar Nitrogen Analyser | Nitrogen is determined by complete combustion of the sample in the presence of oxygen using a Leco nitrogen analyser. The resulting gases pass through various filters to remove interfering gases/particles and nitrous oxide gases are reduced to nitrogen by means of a heated catalyst. An aliquot of the filtered gases is analysed using a thermal conductivity cell, with helium being used as the reference gas and the carrier gas. The output voltage that results is processed by the internal computer and converted to give the nitrogen content of the sample which is then converted into a protein value by calculation using the appropriate conversion factor. |
| Total fat by NMR | AM/C/101 5 | In-house method | The dried sample (obtained via the loss on drying method outlined above) is stabilised at 50oC in a heating block prior to being subjected to pulsed NMR (Nuclear Magnetic Resonance). The resulting oil/fat content is determined automatically by comparing the resonance of the sample with a two point calibration curve constructed using a certified olive oil standard. |
| Ash | AM/C/803 | BS 4401 Part 1 1998 Commission Regulation (EC) 152/2009 | The sample is ignited in an ashing furnace until all organic matter is removed. The weight of residue is expressed as a percentage of the sample weight taken. |
| Total carbohydrate by difference | AM/C/901 | 1169/2011/EEC | Total Carbohydrate is calculated as follows: % Carbohydrate = 100 - (% Moisture + % Fat + % Protein + % Ash) |
| Available carbohydrate by difference | AM/C/901 | 1169/2011/EEC | Available Carbohydrate is calculated as follows: % Carbohydrate = 100 - (% Moisture + % Fat + % Protein + % Ash + % Dietary Fibre) |
| Energy calculations | AM/C/901 | 1169/2011/EEC | Energy is calculated in kcals and in kJ/100g and/or kJ/100ml The calculations performed are as those described in the reference method |
| Dietary fibre (AOAC) | AM/C/309 | AOAC 985.29 | Samples of dried, fat free, foods are gelatinised and treated with heat stable a-amylase and then further digested enzymatically with protease and amyloglucosidase to remove the protein and starch, respectively, present in the sample. IMS is added to precipitate the soluble dietary fibre. The residue is then filtered and washed with IMS and acetone. After drying, the residue is weighed. Sample residues are split and analysed for protein and ash. Total dietary fibre is the weight of the residue less the weight of protein and ash, expressed as a percentage of the sample weight. |
| Total sugar | AM/C/403 | In house based upon Dionex application note 20 | Sugars are extracted in hot water and proteins removed with Carrez solutions. Individual sugars (galactose, glucose, fructose, sucrose, lactose and maltose) are quantified by ion exchange high performance liquid chromatography. The total sugar content of the sample is calculated based upon the summation of the individual quantified sugars detected. The method is suitable for testing food products and pure sugars to determine the individual sugars present. The range of application is 0.01 to 100g/100g for glucose, galactose, fructose and lactose individual sugars and 0.03g/100g for sucrose and maltose. Total sugars are calculated as the sum of the individual sugars. |
| Sodium | AM/C/100 2 | This method has been developed in-house based upon Thermo Fisher application notes and Instrument Manuals | Samples are digested in 50% Nitric Acid and filtered. An Yttrium internal standard is added to the digestion vessel and sodium is quantified by multi-point calibrations on a Thermo icap 7600 Duo Inductively Coupled Plasma – Optical Emission Spectrometer (ICP– OES). |
| Fatty acids including saturdates, mono-saturates and poly-saturdates | AM/C/107 | BS EN ISO 12966-2: 2011 and ISO 5509: 1990 | Fats are converted to free fatty acids by saponification. Fatty acids are converted to their methyl esters by treatment with methanolic acetyl chloride and are then extracted with heptane. Internal standards are employed if an estimation of actual fatty acids present in the fat is required. Identification and quantification of fatty acids is achieved by gas chromatography using flame ionization detection. |

**Supplementary table S2.**

|  |  |  | Species diversity indices | |
| --- | --- | --- | --- | --- |
| Sample | Day of diet collection | Number of reads | Shannon | Simpson |
| Diet 1 | 1 | 25443 | 3.42 | 0.93 |
| Diet 1 | 1 | 24250 | 3.91 | 0.95 |
| Diet 2 | 32 | 17603 | 4.44 | 0.97 |
| Diet 2 | 32 | 31239 | 4.51 | 0.97 |
| Diet 2 | 32 | 19415 | 4.45 | 0.97 |
| Diet 3 | 60 | 23894 | 4.43 | 0.95 |
| Diet 3 | 60 | 15538 | 4.34 | 0.95 |
| Diet 3 | 60 | 6146 | 3.35 | 0.88 |
| Diet 4 | 90 | 17496 | 4.58 | 0.98 |
| Diet 4 | 90 | 17607 | 4.57 | 0.98 |
| Diet 5 | 134 | 21280 | 4.94 | 0.98 |
| Diet 5 | 134 | 18138 | 4.86 | 0.98 |
| Diet 5 | 134 | 14941 | 4.76 | 0.98 |
| Diet 6 | 155 | 14302 | 5.06 | 0.99 |
| Diet 7 | 187 | 14635 | 5.00 | 0.99 |
| Diet 7 | 187 | 16752 | 4.87 | 0.98 |
| Diet 7 | 187 | 18046 | 5.00 | 0.98 |
| Diet 8 | 215 | 20343 | 4.93 | 0.98 |
| Diet 8 | 215 | 19227 | 4.78 | 0.98 |
| Diet 8 | 215 | 2603 | 3.89 | 0.97 |
| Diet 9 | 248 | 16814 | 5.33 | 0.99 |
| Diet 9 | 248 | 19176 | 5.36 | 0.99 |
| Diet 9 | 248 | 20474 | 5.39 | 0.99 |
| Fly 1 | 1 | 21125 | 3.11 | 0.92 |
| Fly 1 | 1 | 26435 | 2.75 | 0.86 |
| Fly 1 | 1 | 25769 | 2.92 | 0.92 |
| Fly 1 | 1 | 25701 | 3.30 | 0.92 |
| Fly 1 | 1 | 30477 | 2.14 | 0.84 |
| Fly 2 | 32 | 23995 | 3.04 | 0.88 |
| Fly 2 | 32 | 34180 | 2.17 | 0.84 |
| Fly 2 | 32 | 20574 | 3.31 | 0.90 |
| Fly 2 | 32 | 18428 | 3.34 | 0.92 |
| Fly 2 | 32 | 22646 | 4.35 | 0.97 |
| Fly 3 | 60 | 26870 | 1.83 | 0.78 |
| Fly 3 | 60 | 19924 | 4.07 | 0.93 |
| Fly 3 | 60 | 31359 | 2.06 | 0.81 |
| Fly 3 | 60 | 27969 | 1.76 | 0.73 |
| Fly 3 | 60 | 23278 | 3.35 | 0.93 |
| Fly 4 | 90 | 19920 | 2.02 | 0.65 |
| Fly 4 | 90 | 24459 | 2.69 | 0.85 |
| Fly 4 | 90 | 9142 | 3.14 | 0.90 |
| Fly 4 | 90 | 5545 | 2.98 | 0.88 |
| Fly 4 | 90 | 31057 | 3.32 | 0.93 |
| Fly 5 | 134 | 20719 | 4.93 | 0.99 |
| Fly 5 | 134 | 27832 | 2.52 | 0.84 |
| Fly 5 | 134 | 11746 | 3.22 | 0.93 |
| Fly 5 | 134 | 28543 | 0.92 | 0.26 |
| Fly 5 | 134 | 1479 | 1.98 | 0.78 |
| Fly 6 | 155 | 32431 | 2.25 | 0.81 |
| Fly 6 | 155 | 27554 | 2.83 | 0.89 |
| Fly 6 | 155 | 18701 | 2.62 | 0.88 |
| Fly 6 | 155 | 28777 | 2.61 | 0.89 |
| Fly 6 | 155 | 31831 | 2.31 | 0.85 |
| Fly 7 | 187 | 35305 | 1.19 | 0.33 |
| Fly 7 | 187 | 33607 | 0.90 | 0.29 |
| Fly 7 | 187 | 30331 | 1.00 | 0.32 |
| Fly 7 | 187 | 34757 | 0.52 | 0.16 |
| Fly 7 | 187 | 26330 | 1.74 | 0.52 |
| Fly 8 | 215 | 25523 | 2.65 | 0.81 |
| Fly 8 | 215 | 21651 | 2.35 | 0.77 |
| Fly 8 | 215 | 22217 | 1.93 | 0.63 |
| Fly 8 | 215 | 20787 | 2.45 | 0.77 |
| Fly 8 | 215 | 20052 | 2.62 | 0.83 |
| Fly 9 | 248 | 21523 | 2.27 | 0.80 |
| Fly 9 | 248 | 19645 | 2.58 | 0.85 |
| Fly 9 | 248 | 28142 | 2.58 | 0.87 |
| Fly 9 | 248 | 24190 | 2.41 | 0.84 |
| Fly 9 | 248 | 33089 | 1.36 | 0.49 |

**Supplementary table S3.**

| **Collection day comparison** | **diff** | **p adj** |
| --- | --- | --- |
| 32-1 | 1.0445 | 0.000508 |
| 60-1 | 1.085864 | 0.00034 |
| 90-1 | 1.315516 | 0.000116 |
| 134-1 | 1.809368 | <0.00001 |
| 155-1 | 2.174232 | <0.00001 |
| 187-1 | 2.242511 | <0.00001 |
| 215-1 | 1.933709 | <0.00001 |
| 248-1 | 2.440366 | <0.00001 |
| 60-32 | 0.041364 | 0.999998 |
| 90-32 | 0.271016 | 0.77756 |
| 134-32 | 0.764869 | 0.003479 |
| 155-32 | 1.129732 | 0.002341 |
| 187-32 | 1.198011 | 0.000034 |
| 215-32 | 0.88921 | 0.000836 |
| 248-32 | 1.395866 | 5.6E-06 |
| 90-60 | 0.229652 | 0.888951 |
| 134-60 | 0.723504 | 0.005667 |
| 155-60 | 1.088368 | 0.003292 |
| 187-60 | 1.156647 | 5.07E-05 |
| 215-60 | 0.847846 | 0.001333 |
| 248-60 | 1.354502 | <0.00001 |
| 134-90 | 0.493853 | 0.153782 |
| 155-90 | 0.858716 | 0.034436 |
| 187-90 | 0.926995 | 0.001651 |
| 215-90 | 0.618194 | 0.043412 |
| 248-90 | 1.12485 | 0.000234 |
| 155-134 | 0.364864 | 0.72142 |
| 187-134 | 0.433143 | 0.168505 |
| 215-134 | 0.124341 | 0.99339 |
| 248-134 | 0.630998 | 0.01711 |
| 187-155 | 0.068279 | 0.999993 |
| 215-155 | -0.24052 | 0.956998 |
| 248-155 | 0.266134 | 0.927089 |
| 215-187 | -0.3088 | 0.525449 |
| 248-187 | 0.197855 | 0.906842 |
| 248-215 | 0.506657 | 0.074502 |

**Supplementary table S4.**

| Diet Samples |  |  | Fly Samples |  |  |
| --- | --- | --- | --- | --- | --- |
| Genus | ASV | r^2^ | Genus | ASV | r^2^ |
| Acetobacter | ASV_5 | -0.54** | Acetobacter | ASV_1 | 0.36* |
| Acetobacter | ASV_1 | 0.63** | Streptomyces | ASV_2 | 0.47** |
| Gluconobacter | ASV_16 | -0.69*** | Acetobacter | ASV_3 | -0.06 |
| Acetobacter | ASV_19 | -0.55** | Acetobacter | ASV_4 | -0.29 |
| Acetobacter | ASV_20 | -0.72*** | Acetobacter | ASV_5 | -0.49*** |
| Brevundimonas | ASV_25 | 0.72*** | Komagataeibacter | ASV_6 | -0.54*** |
| Rhizobium | ASV_24 | -0.30 | Acetobacter | ASV_7 | 0.11 |
| Komagataeibacter | ASV_21 | -0.70*** | Acetobacter | ASV_8 | -0.04 |
| Brevundimonas | ASV_27 | 0.77*** | Gluconacetobacter | ASV_9 | 0.11 |
| Gluconacetobacter | ASV_9 | -0.43* | Acetobacter | ASV_10 | -0.64*** |
| Dysgonomonas | ASV_31 | 0.44* | Acetobacter | ASV_11 | 0.04 |
| Acetobacter | ASV_26 | -0.59** | Acetobacter | ASV_12 | -0.23 |
| Prevotella_9 | ASV_33 | -0.27 | Acetobacter | ASV_13 | -0.60*** |
| Xanthobacter | ASV_30 | 0.72*** | Arthrobacter | ASV_14 | 0.49*** |
| Clostridium | ASV_29 | -0.04 | Shimazuella | ASV_15 | 0.26 |
| Acetobacter | ASV_3 | -0.11 | Gluconobacter | ASV_17 | -0.52*** |
| Lactobacillus | ASV_28 | -0.54** | Asaia | ASV_18 | -0.70*** |
| Lactobacillus | ASV_36 | 0.61** | Rhodococcus | ASV_22 | 0.19 |
| Acetobacter | ASV_35 | -0.30 | Acetobacter | ASV_23 | 0.35* |
| Lactobacillus | ASV_34 | 0.54** | Corynebacterium_1 | ASV_42 | -0.05 |

**Supplementary table S5.**

|  |  |  | **2.5.2014** | **2.6.2014** | **30.6.2014** | **30.7.2014** | **11.9.2014** | **2.10.2014** | **3.11.2014** | **1.12.2014** | **2.1.2015** |
| --- | --- | --- | --- | --- | --- | --- | --- | --- | --- | --- | --- |
| Acetobacter | ASV | 1 |  |  |  | 4 | 5 | 6 | 7 | 8 | 9 |
| Acetobacter | ASV | 3 |  | 2 | 3 | 4 | 5 | 6 | 7 | 8 | 9 |
| Acetobacter | ASV | 4 |  | 2 | 3 | 4 | 5 | 6 | 7 | 8 |  |
| Acetobacter | ASV | 5 | 1 | 2 | 3 | 4 | 5 |  |  | 8 | 9 |
| Komagataeibacter | ASV | 6 | 1 | 2 | 3 |  | 5 |  |  |  |  |
| Acetobacter | ASV | 8 |  |  |  | 4 | 5 | 6 | 7 | 8 | 9 |
| Gluconacetobacter | ASV | 9 | 1 | 2 |  | 4 | 5 | 6 | 7 |  | 9 |
| Acetobacter | ASV | 10 | 1 | 2 | 3 | 4 | 5 | 6 |  |  |  |
| Acetobacter | ASV | 11 |  | 2 |  | 4 | 5 | 6 | 7 | 8 | 9 |
| Acetobacter | ASV | 12 |  | 2 |  | 4 |  |  |  |  |  |
| Gluconobacter | ASV | 16 | 1 | 2 | 3 | 4 |  | 6 |  |  |  |
| Gluconobacter | ASV | 17 | 1 | 2 | 3 |  |  |  |  |  |  |
| Asaia | ASV | 18 | 1 | 2 | 3 | 4 |  |  |  |  |  |
| Acetobacter | ASV | 19 | 1 | 2 | 3 | 4 |  |  |  | 8 | 9 |
| Komagataeibacter | ASV | 21 | 1 |  | 3 |  |  |  |  |  |  |
| Rhizobium | ASV | 24 |  | 2 | 3 |  | 5 | 6 |  |  |  |
| Acetobacter | ASV | 26 | 1 | 2 |  | 4 |  |  |  |  |  |
| Brevundimonas | ASV | 27 |  |  |  |  | 5 |  | 7 | 8 |  |
| Lactobacillus | ASV | 28 | 1 | 2 |  |  | 5 | 6 | 7 |  | 9 |
| Clostridium_sensu_stricto_12 | ASV | 29 |  |  | 3 | 4 | 5 | 6 | 7 |  |  |
| Xanthobacter | ASV | 30 |  |  |  |  | 5 |  | 7 | 8 |  |
| Dysgonomonas | ASV | 31 |  | 2 | 3 |  |  |  |  |  |  |
| SN8 | ASV | 32 | 1 | 2 | 3 | 4 |  |  | 7 | 8 |  |
| Prevotella_9 | ASV | 33 |  | 2 | 3 | 4 |  |  |  |  |  |
| Lactobacillus | ASV | 34 |  | 2 | 3 |  | 5 | 6 |  | 8 |  |
| Acetobacter | ASV | 35 | 1 | 2 | 3 | 4 |  | 6 |  |  |  |
| Lactobacillus | ASV | 36 |  | 2 | 3 |  |  |  | 7 |  |  |
| Falsirhodobacter | ASV | 37 | 1 | 2 | 3 | 4 |  | 6 | 7 | 8 | 9 |
| Acetobacter | ASV | 39 | 1 | 2 |  |  |  | 6 |  |  |  |
| Lactobacillus | ASV | 40 |  | 2 | 3 | 4 | 5 | 6 | 7 | 8 | 9 |
| Rhizobium | ASV | 41 |  |  |  | 4 |  |  |  | 8 | 9 |
| Lactobacillus | ASV | 47 |  | 2 | 3 | 4 | 5 | 6 | 7 |  | 9 |
| Acetobacter | ASV | 48 |  | 2 | 3 |  |  |  |  |  |  |
| Haoranjiania | ASV | 49 |  |  | 3 |  |  |  | 7 |  |  |
| Pseudoxanthomonas | ASV | 51 |  | 2 | 3 |  |  | 6 |  |  |  |
| Uliginosibacterium | ASV | 53 |  |  |  |  |  | 6 | 7 |  |  |
| Gluconobacter | ASV | 57 |  | 2 | 3 | 4 |  |  |  |  |  |
| Lactobacillus | ASV | 58 |  | 2 | 3 | 4 |  |  | 7 | 8 | 9 |
| SN8 | ASV | 59 |  | 2 | 3 |  |  | 6 |  |  |  |
| Pseudoxanthomonas | ASV | 60 |  |  |  | 4 | 5 |  |  |  |  |
| Lactobacillus | ASV | 61 |  |  | 3 | 4 | 5 | 6 | 7 | 8 | 9 |
| Lactobacillus | ASV | 63 | 1 |  |  | 4 | 5 | 6 |  |  |  |
| Comamonas | ASV | 65 | 1 |  | 3 |  |  |  |  |  |  |
| Chryseobacterium | ASV | 66 | 1 | 2 |  |  |  |  | 7 |  |  |
| Acetobacter | ASV | 67 |  | 2 | 3 |  |  |  |  |  |  |
| Prevotella_9 | ASV | 69 |  |  | 3 |  | 5 |  |  |  |  |
| Dysgonomonas | ASV | 72 | 1 | 2 | 3 |  |  |  |  |  |  |
| Paenirhodobacter | ASV | 73 | 1 |  | 3 |  |  |  |  |  |  |
| Acetobacter | ASV | 74 | 1 | 2 |  |  |  |  |  |  |  |
| Lactobacillus | ASV | 75 |  | 2 |  |  |  |  |  | 8 |  |
| Paludibacter | ASV | 76 |  | 2 | 3 |  |  |  |  |  |  |
| Gluconacetobacter | ASV | 81 | 1 | 2 |  |  |  |  |  |  |  |
| Lactobacillus | ASV | 82 | 1 | 2 | 3 |  |  | 6 |  | 8 |  |
| Lactobacillus | ASV | 83 | 1 | 2 |  |  |  | 6 | 7 |  |  |
| Xanthobacter | ASV | 87 |  |  |  |  |  |  | 7 | 8 |  |
| Acetobacter | ASV | 92 |  | 2 | 3 |  |  |  |  |  |  |
| Lactobacillus | ASV | 93 | 1 | 2 | 3 | 4 |  |  |  | 8 |  |
| Chryseobacterium | ASV | 94 | 1 | 2 | 3 |  |  | 6 | 7 | 8 | 9 |
| Rhodovarius | ASV | 98 |  |  |  |  | 5 | 6 | 7 |  | 9 |
| Lactobacillus | ASV | 102 |  | 2 |  | 4 |  |  |  | 8 |  |
| Erysipelothrix | ASV | 105 |  |  | 3 |  | 5 |  |  |  |  |
| Lactobacillus | ASV | 107 | 1 | 2 |  |  |  | 6 |  |  |  |
| Microbacterium | ASV | 109 |  |  |  | 4 | 5 | 6 | 7 | 8 | 9 |
| Luteimonas | ASV | 110 |  | 2 |  | 4 |  |  |  |  |  |
| Terrimonas | ASV | 111 |  | 2 | 3 |  |  |  |  |  |  |
| Xanthobacter | ASV | 114 |  |  |  |  | 5 | 6 |  | 8 |  |
| Reyranella | ASV | 117 |  |  |  |  | 5 |  |  | 8 | 9 |
| Paludibacter | ASV | 120 |  |  | 3 |  |  | 6 |  |  |  |
| Oenococcus | ASV | 121 | 1 | 2 | 3 |  |  |  |  |  |  |
| Lactobacillus | ASV | 122 |  | 2 |  | 4 | 5 |  | 7 | 8 | 9 |
| Lactobacillus | ASV | 123 |  | 2 |  | 4 |  |  |  |  |  |
| Flavobacterium | ASV | 126 |  |  | 3 | 4 |  |  |  |  |  |
| Diaminobutyricimonas | ASV | 129 |  |  | 3 |  | 5 |  |  |  |  |
| Variovorax | ASV | 134 |  |  |  |  |  | 6 |  |  | 9 |
| Lactobacillus | ASV | 135 | 1 |  | 3 |  |  |  |  |  |  |
| Rhizobium | ASV | 137 |  | 2 | 3 |  |  |  | 7 |  |  |
| Lactobacillus | ASV | 142 |  | 2 |  |  |  | 6 |  |  |  |
| Haoranjiania | ASV | 144 |  | 2 | 3 | 4 |  |  | 7 |  |  |
| Chryseobacterium | ASV | 145 | 1 |  | 3 |  |  |  |  |  |  |
| Clostridium_sensu_stricto_12 | ASV | 146 |  | 2 | 3 | 4 | 5 |  |  |  | 9 |
| Bacteroides | ASV | 147 |  |  |  | 4 |  |  |  |  |  |
| Lactobacillus | ASV | 155 | 1 | 2 | 3 |  |  |  |  | 8 |  |
| Lactobacillus | ASV | 156 |  |  |  |  | 5 | 6 |  |  |  |
| Lactobacillus | ASV | 157 | 1 |  | 3 |  |  | 6 |  |  |  |
| Lactobacillus | ASV | 161 |  | 2 |  |  |  |  | 7 |  |  |
| Lactobacillus | ASV | 162 |  | 2 |  |  | 5 |  |  |  |  |
| Shinella | ASV | 164 |  |  | 3 | 4 |  |  | 7 |  |  |
| Lactobacillus | ASV | 167 | 1 |  | 3 |  |  |  |  |  |  |
| Ancylobacter | ASV | 172 |  |  |  |  | 5 | 6 | 7 | 8 |  |
| Lactobacillus | ASV | 173 | 1 |  | 3 |  |  |  |  |  |  |
| Lactobacillus | ASV | 180 |  |  |  |  | 5 | 6 |  | 8 |  |
| Afipia | ASV | 181 |  |  |  | 4 | 5 |  | 7 | 8 |  |
| Paludisphaera | ASV | 183 |  |  |  |  | 5 | 6 | 7 | 8 |  |
| Clostridium_sensu_stricto_19 | ASV | 188 |  |  |  |  |  |  | 7 |  | 9 |
| Sphingobacterium | ASV | 192 | 1 |  |  |  |  |  | 7 |  |  |
| Pleomorphomonas | ASV | 194 |  |  |  |  | 5 |  |  | 8 |  |
| Lactobacillus | ASV | 217 |  |  |  | 4 |  |  | 7 |  |  |
| Lactobacillus | ASV | 221 |  |  |  |  | 5 |  | 7 |  |  |
| Reyranella | ASV | 228 |  |  |  |  | 5 |  |  |  |  |
| Lactobacillus | ASV | 233 |  |  |  |  | 5 |  | 7 |  |  |
| Lactobacillus | ASV | 235 |  |  |  | 4 |  |  |  | 8 |  |
| Stenotrophomonas | ASV | 247 | 1 |  | 3 |  |  |  |  |  |  |
| Mesorhizobium | ASV | 256 |  |  |  |  |  |  | 7 | 8 |  |
| Lactobacillus | ASV | 300 | 1 |  |  |  |  |  |  | 8 |  |
| Clostridium_sensu_stricto_1 | ASV | 321 |  |  |  |  |  |  | 7 | 8 |  |
| Sphingobacterium | ASV | 322 | 1 |  |  |  |  |  | 7 |  |  |
| Erysipelothrix | ASV | 371 |  |  |  |  |  |  | 7 | 8 | 9 |
| Sphingobacterium | ASV | 404 | 1 |  |  |  |  |  | 7 |  |  |
| Ancylobacter | ASV | 518 |  |  |  | 4 | 5 |  | 7 |  |  |
| Lactobacillus | ASV | 1008 |  |  |  |  | 5 |  | 7 |  |  |

**Supplementary table S6.**

| **Collection day comparison** | **diff** | **p adj** |
| --- | --- | --- |
| 32-1 | 0.696429 | < 0.00001 |
| 60-1 | -1.38889 | < 0.00001 |
| 90-1 | -1.64583 | < 0.00001 |
| 134-1 | -2.34615 | < 0.00001 |
| 155-1 | -3 | < 0.00001 |
| 187-1 | -2.83333 | < 0.00001 |
| 215-1 | -2.81818 | < 0.00001 |
| 248-1 | -2.5 | < 0.00001 |
| 60-32 | -2.08532 | < 0.00001 |
| 90-32 | -2.34226 | < 0.00001 |
| 134-32 | -3.04258 | < 0.00001 |
| 155-32 | -3.69643 | < 0.00001 |
| 187-32 | -3.52976 | < 0.00001 |
| 215-32 | -3.51461 | < 0.00001 |
| 248-32 | -3.19643 | < 0.00001 |
| 90-60 | -0.25694 | 0.725827 |
| 134-60 | -0.95726 | < 0.00001 |
| 155-60 | -1.61111 | < 0.00001 |
| 187-60 | -1.44444 | < 0.00001 |
| 215-60 | -1.42929 | < 0.00001 |
| 248-60 | -1.11111 | < 0.00001 |
| 134-90 | -0.70032 | < 0.00001 |
| 155-90 | -1.35417 | < 0.00001 |
| 187-90 | -1.1875 | < 0.00001 |
| 215-90 | -1.17235 | < 0.00001 |
| 248-90 | -0.85417 | < 0.00001 |
| 155-134 | -0.65385 | 0.000257 |
| 187-134 | -0.48718 | 0.031817 |
| 215-134 | -0.47203 | 0.024796 |
| 248-134 | -0.15385 | 0.96603 |
| 187-155 | 0.166667 | 0.972596 |
| 215-155 | 0.181818 | 0.938289 |
| 248-155 | 0.5 | 0.016462 |
| 215-187 | 0.015152 | 1 |
| 248-187 | 0.333333 | 0.385713 |
| 248-215 | 0.318182 | 0.374205 |

**Supplementary Figure S1.**

**
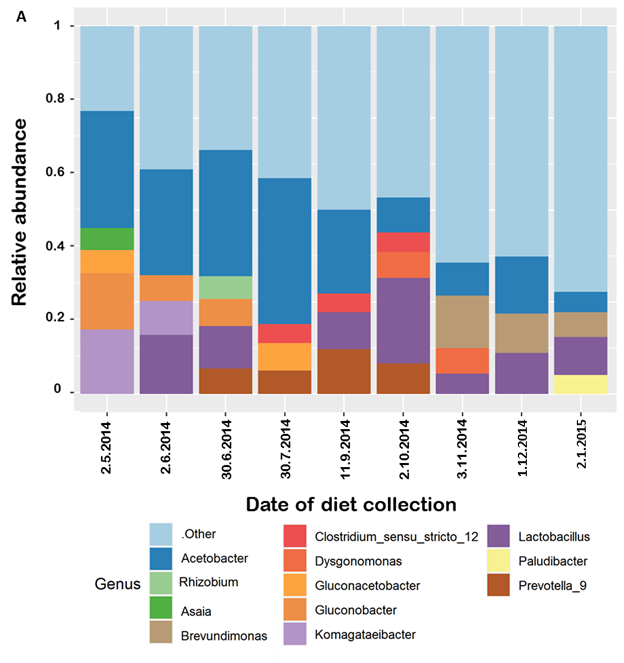
**

**
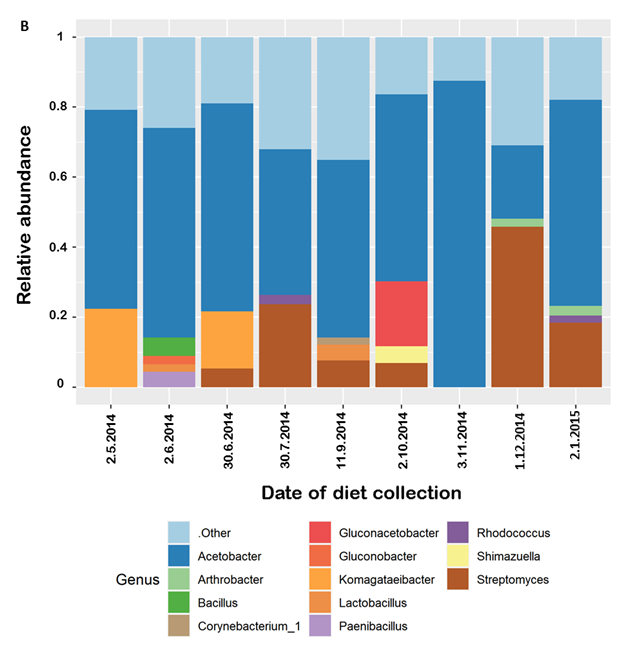
**

**Supplementary Figure S2.**


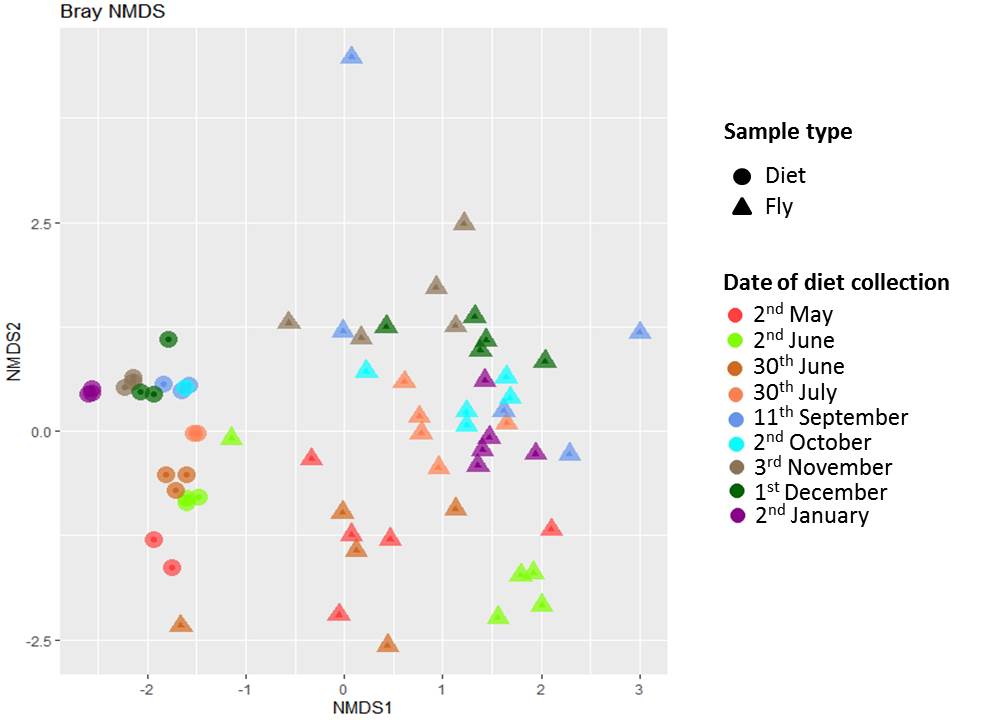


**Supplementary Figure S3.**


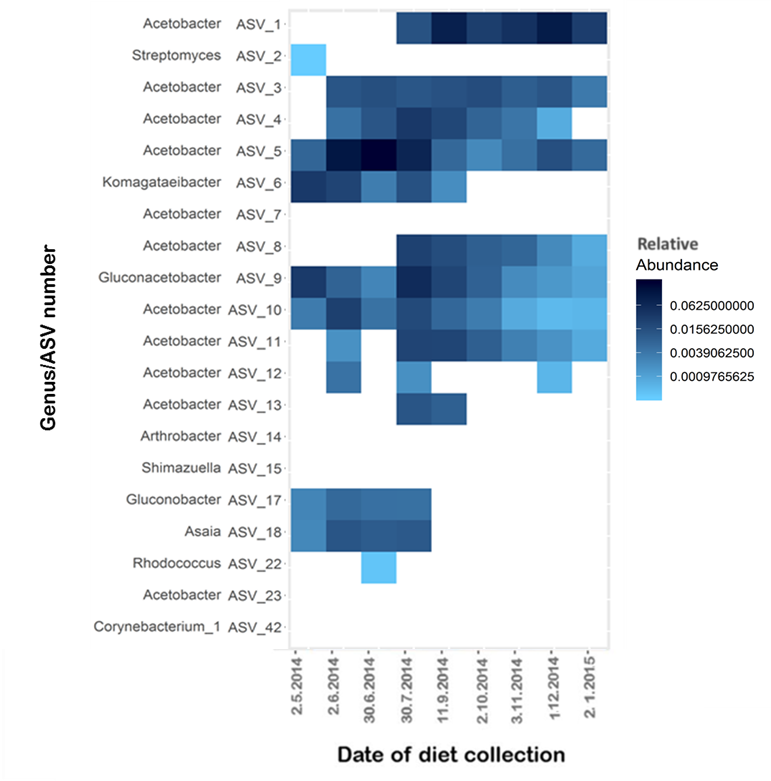

Supplement: Supplementary file 1 — Supplementary Information. [file 41598_2021_98119_MOESM1_ESM.docx]
